# MRE11 and ATM Expression Levels Predict Rectal Cancer Survival and Their Association with Radiotherapy Response

**DOI:** 10.1371/journal.pone.0167675

**Published:** 2016-12-08

**Authors:** Vincent Ho, Liping Chung, Maxine Revoltar, Stephanie H. Lim, Thein-Ga Tut, Askar Abubakar, Chris J. Henderson, Wei Chua, Weng Ng, Mark Lee, Paul De Souza, Matthew Morgan, C. Soon Lee, Joo-Shik Shin

**Affiliations:** 1 School of Medicine, Western Sydney University, Penrith, New South Wales, Australia; 2 Ingham Institute for Applied Medical Research, Liverpool, New South Wales, Australia; 3 Department of Medical Oncology, Liverpool Hospital, Liverpool, New South Wales, Australia; 4 South Western Sydney Clinical School, University of New South Wales, Liverpool, New South Wales, Australia; 5 Department of Anatomical Pathology, Liverpool Hospital, Liverpool, New South Wales, Australia; 6 Department of Radiation Oncology, Liverpool Hospital, Liverpool, New South Wales, Australia; 7 Discipline of Medical Oncology, School of Medicine, Western Sydney University, Liverpool, New South Wales, Australia; 8 Department of Surgery, Bankstown Hospital, Bankstown, New South Wales, Australia; 9 Discipline of Pathology, School of Medicine, Western Sydney University, Campbelltown, New South Wales, Australia; Taipei Medical University, TAIWAN

## Abstract

**Background:**

Aberrant expression of DNA repair proteins is associated with poor survival in cancer patients. We investigated the combined expression of MRE11 and ATM as a predictive marker of response to radiotherapy in rectal cancer.

**Methods:**

MRE11 and ATM expression were examined in tumor samples from 262 rectal cancer patients who underwent surgery for rectal cancer, including a sub-cohort of 54 patients who were treated with neoadjuvant radiotherapy. The relationship between expression of the two-protein panel and tumor regression grade (TRG) was assessed by Mann–Whitney U test and receiver operating characteristics area under curve (ROC-AUC) analysis. The association between expression of the two-protein panel and clinicopathologic variables and survival was examined by Kaplan-Meier methods and Cox regression analysis.

**Results:**

A high score for two-protein combined expression in the tumor center (TC) was significantly associated with worse disease-free survival (DFS) (*P* = 0.035) and overall survival (OS) (*P* = 0.003) in the whole cohort, and with DFS (*P* = 0.028) and OS (*P* = 0.024) in the neoadjuvant subgroup (n = 54). In multivariate analysis, the two-protein combination panel (HR = 2.178, 95% CI 1.115–4.256, *P* = 0.023) and perineural invasion (HR = 2.183, 95% CI 1.222–3.899, *P* = 0.008) were significantly associated with DFS. Using ROC-AUC analysis of good versus poor histological tumor response among patients treated preoperatively with radiotherapy, the average ROC-AUC was 0.745 for the combined panel, 0.618 for ATM alone, and 0.711 for MRE11 alone.

**Conclusions:**

The MRE11/ATM two-protein panel developed in this study may have clinical value as a predictive marker of tumor response to neoadjuvant radiotherapy, and a prognostic marker for disease-free and overall survival.

## Introduction

Colorectal cancer (CRC) is the third leading cause of cancer related death worldwide [1]. It is becoming increasingly important to identify prognostic and predictive markers for rectal cancer. Surgical resection remains the definitive treatment for CRC. However, rectal cancers are more challenging to resect than their colonic counterparts due to limited access within the pelvic space, as well as close proximity to the mesorectal fascia and pelvic organs. As a result, patients with rectal cancer consistently suffer from inferior survival outcomes relative to colon cancer patients [2]. Preoperative radiotherapy aims to downstage the primary tumor, eradicate microscopic disease, and reduce recurrence rates [3–5]. Unfortunately, tumor response to radiotherapy varies between individuals, even after adjusting for clinico-histopathological variables. Tumor down staging following preoperative radiotherapy occurs in approximately 60% of patients, but only 10–30% will display a complete response [6] [7]. The availability of a predictive marker of radiation sensitivity would enable selective administration of therapy to those most likely to respond.

Radiotherapy instigates cell death by causing ionizing radiation-induced double-strand breaks (DSBs) in the DNA, which initiates the DNA damage response (DDR) leading to cell cycle arrest and repair of the damage or, if repair is unsuccessful, cell death. Two early, integral components in this pathway are ataxia telangiectasia mutated (ATM), a serine/threonine protein kinase belonging to the phosphatidylinositol 3-kinase-like (PIKK) family [8] and the MRN complex, a trimer of MRE11 (meiotic recombination 11), RAD50 and NBS1 molecules. The latter complex binds to the DSBs and generates regions of single stranded DNA with its nuclease activity, which then recruits ATM to the site of genetic damage. ATM in turn affects cell cycle arrest [9].

As might be expected from the critical role of DNA repair in maintaining genomic integrity, many cancers, including CRC, exhibit deficiencies in the DNA damage response and DNA repair pathways. Specifically, MRE11 deficiency is associated with improved overall survival (OS) and long-term disease-free survival (DFS) in patients with stage III colon cancer independent of treatment [10], suggesting that MRE11 status has value as a prognostic marker in CRC.

We hypothesized that acquired deficiencies in ATM and/or MRE11 would lead to a dysfunctional repair mechanism and an inability to restore genomic stability, resulting in increased tumor cell death following radiotherapy. At least in the relatively small, but well characterized subgroup of microsatellite unstable CRCs with deficiencies in DNA mismatch repair (MMR) proteins, the accumulation of errors during replication and recombination [11] presents a mechanism for inactivation of ATM and MRE11 through function-ablating mutations. As a corollary, we proposed that ATM and MRE11 might be used as a predictive marker of radiosensitivity in rectal cancer patients, which would correlate with improved patient outcomes.

Thus, we investigated the value of expressions of MRE11, ATM and combined MRE11/ATM as a predictive marker of response to radiotherapy in rectal cancer and as a prognostic marker in general, and performed univariate and multivariate analyses on various clinicopathologic factors according to their expression levels.

## Materials and Methods

### Patient samples

This study received ethics approval from the South Western Sydney Local Health District (SWSLHD) Human Research Ethics Committee (reference number HREC/12/LPOOL/102). Specifically, the research was conducted under the Protocol numbers X01-0138 and X03-0291 for the IHC assessment in paraffin embedded rectal cancer tissues and retrospective survival analyses. The ethics committee waived the need for direct consent for patients as samples and clinical data were all de-identified before access.

Specimens were obtained from 262 rectal cancer patients who underwent surgery in the SWLHD from 2000–2011. Clinicohistopathological data were collated and are summarized in Table 1. Standard radiotherapy treatments were at a dose of 25 Gy in 5 treatment fractions when performed alone or at 50.4 Gy in 28 treatment fractions when combined with 5 fluoro-uracil based chemotherapy. Short-term response to radiotherapy was measured by tumor regression grade (TRG) per the 7^th^ edition of the American Joint Committee on Cancer (AJCC) manual [12] on a scale of 0 to 3: 0, complete response with no viable malignant cells; 1, moderate response with single or small groups of malignant cells; 2, minimal response with residual malignancy outgrown by fibrosis; 3, poor response with extensive residual malignancy. TRG 0, 1 and 2 were categorized as responders and TRG 3 as non-responders. Long-term response to radiotherapy was measured by survival outcomes including DFS and OS. Specimens obtained from primary surgery for rectal or rectosigmoid cancers were obtained from the South-Western Area Pathology database, Australia. Surgery consisted of total mesorectal excision, with anterior or abdominoperineal resection. Variables of interest included age, gender, pathological TNM stage, grade, vascular invasion, perineural invasion, tumor-infiltrating lymphocytes, and treatment. Outcomes of interest were DFS, OS and histologic TRG in the resected bowel for cases treated with neoadjuvant chemoradiation. DFS was defined as the time from diagnosis to first recurrence, if any. OS was defined as the time from diagnosis to last follow-up or death. Follow-up consisted of regular clinic visits, colonoscopy, blood tests, and imaging at the discretion of the treating specialist.

**Table 1:** Patient characteristics.

	All Patients (%)	Preoperative Radiotherapy Group
**Total, n**	262	54
**Age median**	71	67
**Gender**		
**Male**	174 (66.4)	38 (70.4)
**Female**	88 (33.6)	16 (29.6)
**Tumor stage**		
**T1-2**	86/257 (33.5)	17/54 (31.5)
**T3-4**	171/257 (66.5)	37/54 (68.5)
**Node stage**		
**N0**	137/256 (53.5)	28/54 (51.9)
**N1-2**	118/256 (46.5)	26/54 (48.1)
**Metastasis stage**		
**M0**	220/237 (92.8)	52/53 (98.1)
**M1**	17/237 (7.2)	1/53 (1.9)
**Grade**		
**1–2**	242/262 (92.4)	50/54 (92.6)
**3**	20/262 (7.6)	4/54 (7.4)
**Vascular invasion**		
**Absent**	198/260 (76.2)	46/54 (85.2)
**Present**	62/260 (23.8)	8/54 (14.8)
**Perineural invasion**		
**Absent**	218/260 (83.8)	41/54 (75.9)
**Present**	42/260 (16.2)	13/54 (24.1)
**Radiotherapy**		
**Total**	76/245 (31.0)	-
**Neoadjuvant**	54/76 (71.1)	-
**Adjuvant**	22/76 (28.9)	0/54 (0)
**Recurrence**		
**Absent**	129/211 (61.1)	48/54 (88.9)
**Present**	82/211 (38.9)*	6/54 (11.1)†
**Tumor regression grade**		
**0–2 (good response)**	-	9/54 (16.7)
**3 (poor response)**	-	45/54 (83.3)
**Chemotherapy**		
**Total**	98/219 (44.7)	38/54 (70.4)
**Neoadjuvant**	38/98 (38.8)	31/38 (81.6)
**Adjuvant**	60/98 (61.2)	7/38 (18.4)

* Local and distant recurrence,

^†^ Local recurrence only.

### Sample preparation and tissue microarrays

For each patient, donor blocks of archived paraffin-embedded tissue were retrieved and two 1mm diameter cores were obtained from each sampling site: tumor center (TC), tumor periphery at invasive edge (TP), normal mucosa close/adjacent to tumor, normal mucosa well away from tumor, usually at the resection end margin, and from involved lymph nodes (LN). These were transferred into pre-drilled wells in a tissue microarray block and mounted on slides for immunohistochemistry.

### Immunohistochemistry

Slides were deparaffinized in xylene and rehydrated in graded ethanol. Antigen retrieval was performed with Envision^™^ FLEX Target Retrieval Solution, pH 9.0, in a 98°C water bath for 10 minutes for ATM and 45 minutes for MRE11. This was followed by incubation for 5 minutes at room temperature with Envision^™^ FLEX Peroxidase-Blocking Reagent to block endogenous peroxidases. Slides were incubated with monoclonal anti-ATM primary antibody [2C1 (1A1)] (1:800 dilution, Abcam, Cambridge, UK) for 30 minutes at room temperature and with anti-MRE11 primary monoclonal mouse antibody (1:600 dilution, ab214, Abcam). After washing with TBST buffer, slides were incubated for 15 minutes with Dako mouse linker, rinsed, and incubated for 30 minutes with anti-mouse secondary antibody. The peroxidase substrate used was a mixture of Envision^™^ FLEX DAB+Chromogen DM827 and Envision^™^ FLEX Substrate Buffer DM823 (DAKO). Slides were counterstained with hematoxylin, washed with cold water, and then dipped 10 times in Scott Bluing solution. Slides were immediately rinsed with cold water before dehydration and mounting.

### Immunohistochemical scoring

ATM expression was initially scored as the product of percentage and intensity of staining based on Angele et al [13]. The percentage of positive staining was scored as: 1, <25%; 2, 25%–50%; 3, 50%–75%; or 4,>75%. The intensity of staining was graded as: null, 0; low, 1; moderate, 3; or high, 5. The two scores were multiplied to create a composite score between 0 and 20 and analyzed against clinicohistopathological and clinical outcome data. The percentage of MRE11-positive cells and staining intensity were also scored using the methods published previously [14]. Intensity was graded as: negative, 0; weak, 1; moderate, 2; or strong, 3. The percentage of positive cells was graded as: 0, <5%; 1, 5%–25%; 2, 26%–50%; 3, 51%–75%; and 4, >75%. These two measures were multiplied to give weighted scores ranging from 0–12. To combine the two proteins as a biomarker panel, all cases were categorized into either a low expression group (score range: 0–5) and a high expression group (score range: 6–32).

### Statistical analysis

Statistical analysis was performed with SPSS Statistics for Windows 20.0 (Chicago, IL, USA). ATM expression was compared with clinicohistopathological data using Pearson’s χ^2^ test, and the association of MRE11 expression with clinicohistopathological variables was assessed by Fisher's exact test. ATM and MRE11 expression levels were compared and combined by binary logistic regression as described previously [15]. Survival analyses were performed in the overall cohort and separately in patients who received preoperative radiotherapy. Univariate and multivariate analyses were performed using Kaplan Meir curves and Cox’s proportional hazards survival modeling for the combined two-marker expression levels from cancer core and periphery samples. Covariates were sex, age, TNM stage, grade, vascular invasion, perineural invasion, treatment with chemotherapy and radiotherapy, and TRG. Univariate analysis by the Mann–Whitney U test was also used to assess associations between the single and combined two-marker expression levels in rectal tumor tissue with TRG, which was further characterized with receiver operating characteristic—area under curve (ROC-AUC) analysis. *P*<0.05 was considered statistically significant.

## Results

### Patient populations

Patient characteristics are detailed in Table 1. The patients’ median age was 71 years (range: 35–100 years). Among 262 patients, 174 (66.4%) were male and 88 (33.6%) were female. Seventy six of 245 (31.0%) patients were treated with radiotherapy, of which 54 of 76 (71.1%) received preoperative therapy. Disease-free and OS data were available for 211 and 248 patients, respectively. Patients were followed for a median period of 3.2 years (range: 0−12.6 years). Local recurrence of disease occurred in 82/211 (38.9%) patients. The median time to recurrence was 2.12 years. At the time of the study, 141/248 (56.9%) patients were alive. The median time to death was 2.5 years following surgery (range: 0−11.1 years). Among patients who received preoperative radiotherapy, local recurrence occurred in 6/54 (11.1%) patients and median time to recurrence was 2.61 years (range: 0.75−4.29 years). All six patients (100%) had died by the end of this study. The median time to death following recurrence was 3.81 years (range: 0.6−10.9 years).

### Establishment of the combined two-protein biomarker panel

ATM and MRE11 protein expression levels in the TC were tested in a forward and reverse binary logistic regression analysis using a data set of immunohistochemical scoring derived from 257 tumor samples and 255 normal tissues. The final biomarker model gave an average receiver operator characteristic area under the curve (ROC-AUC) value of 0.849 for the combination of the two proteins. Similarly, ATM and MRE11 protein expression levels in the TP (tumor, n = 258; normal, n = 255) were also tested, and the model gave an average ROC-AUC value of 0.837. The sensitivity and specificity of the two-protein combined panel were 80.9% and 70.3% for TC and 61.6% and 48.8% for TP, respectively, and overall accuracy was 75.6% (TC) and 55.2% (TP) (S1 Table).

### Relationship between ATM and MRE11 protein expression and clinicopathological features

We examined the association of ATM and MRE11 expression levels with clinicopathological variables independently. ATM protein expression was analyzed in 259 central and 260 peripheral tumor cores. Samples from TC displayed high expression in 84% and low expression in 16%. Samples from TP showed high expression in 79% and low expression in 21%. There was a higher level of ATM expression in TC (mean (M) = 6.84) compared to TP (M = 5.28). This difference was found to be significant (P<0.001).

ATM expression was seen in 199/228 (87%) of adjacent normal mucosa and 204/250 (82%) of distal normal mucosa. ATM expression was higher in normal mucosa taken near the tumor (M = 6.43) compared to samples taken away from the tumor (M = 4.25). Again, this finding was statistically significant (P<0.001). ATM expression in both TC and TP were significantly higher than in distal normal mucosa (P<0.001 and P = 0.005 respectively), but not significantly different to that in the adjacent normal mucosa.

Low ATM expression in the TP was associated with older age (*P* = 0.013) and higher histologic grade (*P* = 0.044).(S2 Table). There was no correlation with sex, TNM category, vascular invasion, or perineural invasion. Expression of ATM in TC was not associated with any clinicohistopathological variables.

MRE11 expression in the TC was high in 45% of cases and low in 52%. Expression in the TP was high in 56% and low in 44%. Mean weighted scores were significantly different between TC and TP (5.5 versus 5.8, *P*<0.05 by paired *t*-test). The mean MRE11 score was 4.2 for both adjacent and distal normal tissue, which was significantly different from the score for both the TC and TP (*P*<0.001). There were no significant associations between MRE11 score in the TC or TP and clinicopathologic characteristics (S3 Table).

### Association between combined expression levels with pathological variables and prognosis

**Fig 1:**
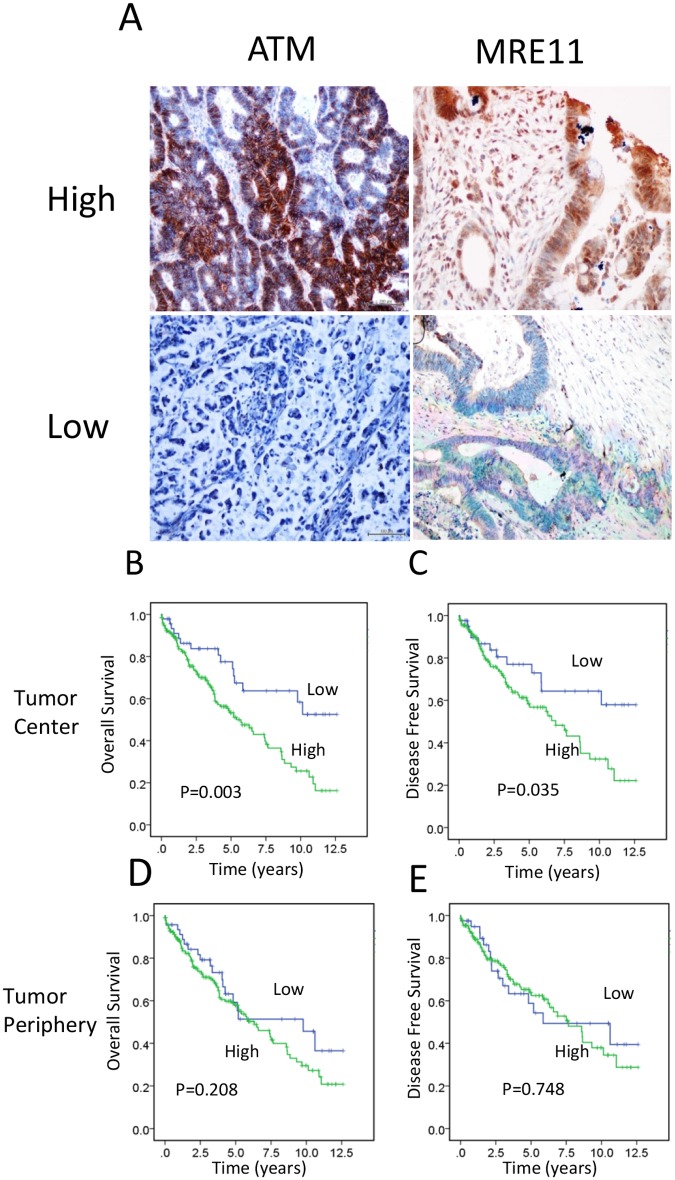
Association between combined protein expression levels of ATM and MRE11 in rectal cancer tissues and survival. (A) Representative immunohistochemical staining of ATM and MRE11 in rectal cancer tissues. Bar = 200 μm. Staining for each protein was scored as high or low, as described in the Materials and Methods. (B) Overall survival according to combined protein expression levels of ATM and MRE11 was determined by Kaplan−Meier survival analyses and compared using the log-rank test. Patients with high combined protein expression levels of ATM and MRE11 in the TC (green line) showed significantly worse OS than those with low expression (blue line; *P* = 0.003). (C) Similarly, patients with high expression levels in the TC (green line) exhibited worse disease-free survival than those with low expression (blue line; *P* = 0.035). (D and E) When measured in the TP, no significant survival difference was seen between the high- and low-expression groups for overall survival (*P* = 0.208) or disease-free survival (*P* = 0.748).

Next, we examined a possible association of clinicopathological characteristics and survival outcomes with the ATM/MRE11 two-protein combined panel. Fig 1A shows representative immunohistochemical staining of high and low/absent ATM and MRE11 expression in rectal cancer tissues. Results from the combined marker analysis differed from our initial single biomarker studies of ATM and MRE11 alone, in that a high combined expression of ATM and MRE11 was significantly associated with a number of clinicopathological variables. These included neoadjuvant status (*P* = 0.001 for TC), TRG (*P* = 0.03 for TC, *P* = 0.011 for TP), age (*P* = 0.02 for TP), and nodal stage (*P* = 0.042 for TP) (Table 2).

**Table 2:** Associations between the combined expression of ATM and MRE11 in the tumor center and tumor periphery and clinicohistopathological data.

		Combined TC			Combine TP		
		Low (%)	High (%)	P value	Low (%)	High (%)	P value
**Sex**	Male	62.5	67.3	0.562	65.3	66.8	0.381
	Female	37.5	32.7		34.7	33.2	
**Age**	≤70	43.8	46.3	0.747	42.9	46.0	**0.020**
	>70	56.2	53.7		57.1	54.0	
**Tumor stage**	T1–2	50.0	29.5	0.496	38.3	31.9	0.877
	T3–4	50.0	70.5		61.7	68.1	
**Node stage**	Negative	60.0	52.4	0.840	44.7	55.8	**0.042**
	Positive	40.0	47.6		55.3	44.2	
**Metastasis stage**	M0	97.8	91.6	0.282	100	91.1	0.252
	M1	2.2	8.4		0	8.9	
**Grade**	1–2	93.8	92.1	0.508	89.8	92.9	0.131
	3	6.2	7.9		10.2	7.1	
**Vascular invasion**	Absent	82.6	74.6	0.231	77.1	75.6	0.380
	Present	17.4	25.4		22.9	24.4	
**Perineural invasion**	Absent	84.8	83.6	0.928	85.4	83.3	0.500
	Present	15.2	16.4		14.6	16.7	
**Adjuvant therapy**	No	70.0	69.5	0.483	65.0	70.9	0.55
	Yes	30.0	30.5		35.0	29.1	
**Neoadjuvant therapy**	No	65.2	81.1	**0.001**	71.7	79.9	0.64
	Yes	34.8	18.9		28.3	20.1	
**Tumor regression grade**	0–2	31.6	8.0	**0.030**	33.3	6.0	**0.011**
	3	68.4	92.0		66.7	94.0	

Abbreviations: TC, Tumor center; TP, Tumor periphery

In Kaplan Meier survival analysis, a high score for the two-protein combined expression in the TC was significantly associated with worse OS (*P* = 0.003) and DFS (*P* = 0.035) (Fig 1B and 1C). However, no significant survival difference was seen between high and low groups for expression in the TP (OS, *P* = 0.208 and DFS, *P* = 0.748; Fig 1D and 1E, respectively). In univariate Cox regression analysis, a high two-protein combined status in the TC (combined TC high versus low: HR = 1.944, 95% CI 0.037–3.645, *P* = 0.038) was significantly associated with reduced DFS (Table 3). In multivariate Cox analysis (adjusted for combined expression of ATM and MRE11, and perineural invasion), the two-protein combination panel (HR = 2.178, 95% CI 1.115–4.256, *P* = 0.023) as well as perineural invasion (HR = 2.183, 95% CI 1.222–3.899, *P* = 0.008) remained significantly associated with DFS (Table 3).

**Table 3:** Cox regression analyses of combined TC expression level with clinicopathologic variables and DFS.

		Univariate			Multivariate		
	n (%)	HR	95% CI	P Value	HR	95%	P Value
**Combined TC**							
**High**	81.3	1.944	1.037–3.645	**0.038**	2.178	1.115–4.256	**0.023**
**Low**	18.7						
**Tumor stage**							
**T1–2**	33.5	1.504	0.899–2.517	0.12			
**T3–4**	66.5						
**Node stage**							
**Negative**	53.5	1.132	0.703–1.825	0.609			
**Positive**	46.5						
**Grade**							
**1–2**	92.4	1.617	0.699–3.739	0.261			
**3**	7.6						
**Vascular invasion**							
**Absent**	76.2	1.164	0.637–2.129	0.662			
**Present**	23.8						
**Perineural invasion**							
**Absent**	83.8	2.334	1.310–4.157	**0.004**	2.183	1.222–3.899	**0.008**
**Present**	16.2						
**Adjuvant therapy**							
**No**	91.0	0.605	0.343–1.067	0.082			
**Yes**	9.0						
**Neoadjuvant therapy**							
**No**	77.7	1.088	0.630–1.878	0.762			
**Yes**	22.3						
**LN-negative**† **combined TC**	53.5				1.343	0.591–3.052	0.481
**LN-positive**† **combined TC**	46.5				3.474	1.054–11.451	**0.041**

Abbreviations: HR, hazard ratio; CI, confidence intervals; TC, tumor center; LN, lymph node;

^†^denotes interaction

### Prognostic implications of two-protein combined panel in LN-positive and neoadjuvant radiotherapy subgroups

**Fig 2:**
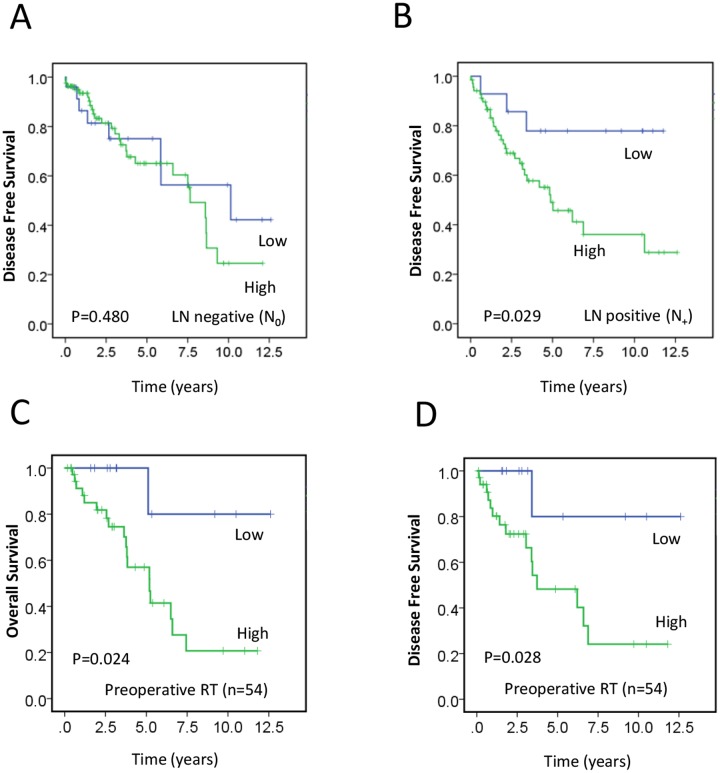
Kaplan-Meier survival analysis. (A) and (B) respectively show survival curves of high (green line) and low (blue line) ATM/MRE11 two-protein expression groups in lymph node (LN) negative and LN positive rectal cancers. These show the effect of LN status on the association between expression levels and disease-free survival (DFS). (C) and (D) respectively show overall survival (OS) and DFS survival curves of 54 patients who received preoperative radiotherapy in terms of high (green line) and low (blue line) two protein expression groups. Worse OS (*P* = 0.024) and DFS (*P* = 0.028) were seen with high expression.

As noted above, DFS of rectal cancer patients with overexpression of the two-protein combined panel was significantly worse than that of patients with lower expression. When patients were grouped according to LN involvement, high expression of the two-protein combined panel was associated with decreased DFS in patients with LN-positive tumors (*P* = 0.029; Fig 2B) but not in those with LN-negative tumors (*P* = 0.480; Fig 2A). By multivariate Cox analysis, expression of the two-protein combined panel in TC in the LN positive subgroup significantly correlated with DFS (HR = 3.474, 95% CI 1.054–11.451, *P* = 0.041) (Table 3).

The OS estimates in the subgroup with neo-adjuvant radiotherapy are shown in Fig 2C, demonstrating that higher combined expression level was significantly associated with worse OS (*P* = 0.024). Similarly, DFS of radiotherapied patients with overexpression of two-protein combined panel was significantly worse than that of patients with lower expression (Fig 2D, *P* = 0.028), implicating that the MRE11/ATM two-protein panel has specific potential as a predictive marker of tumour response to radiotherapy.

### ATM and MRE expression in relation to TRG

**Fig 3:**
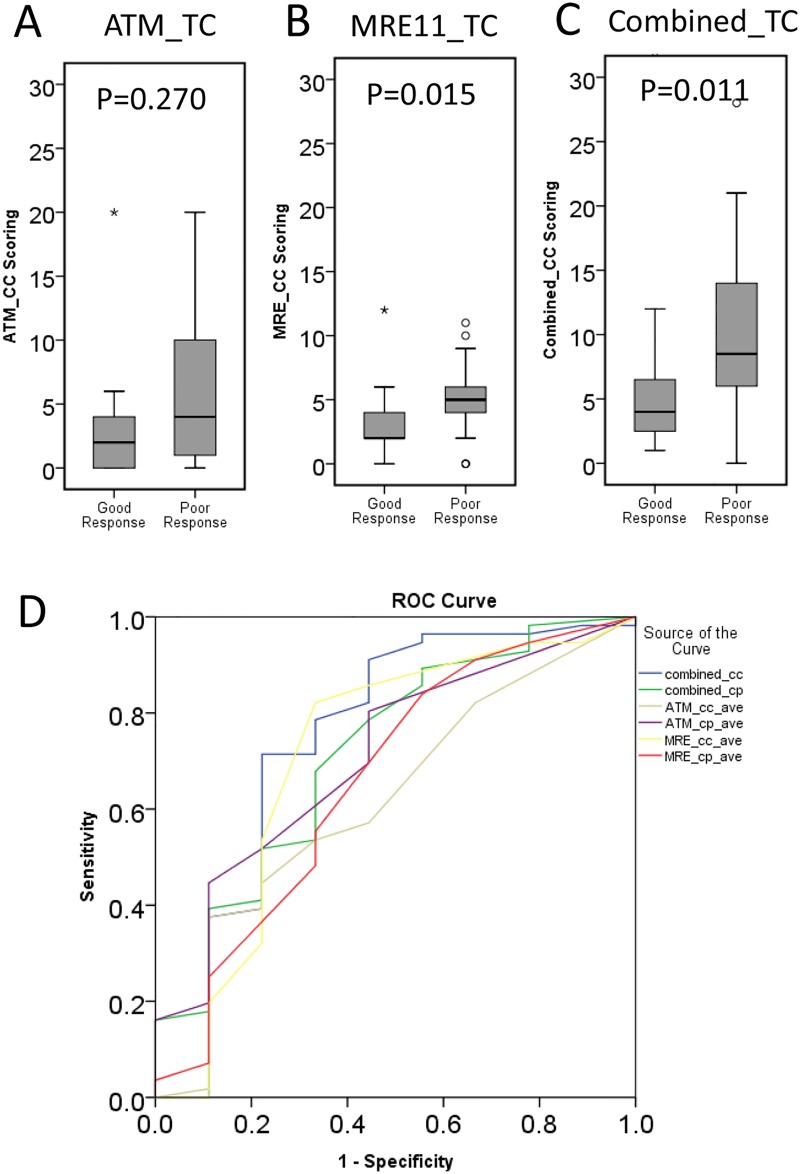
ROC-AUC analysis of ATM, MRE11, and combined protein panel expressions with tumour regression grade (TRG). (A−C) Box plots show levels of ATM, MRE11, and their combined expression in the TC categorized by TRG as 0–2 (good response) or 3 (poor response). The association between protein expression with TRG was examined by Mann−Whitney U test (ATM, *P* = 0.27; MRE11, *P* = 0.015; and combined proteins, *P* = 0.011). (D) Receiver operating characteristic (ROC) curve analysis comparing the performance of ATM and MRE11 alone with the combined 2-protein panel.

To investigate the relationship between TRG and tissue levels of ATM, MRE11, and the two-protein combined panel, univariate analysis was carried out using the Mann−Whiney U test (SPSS). A significant association was seen between increasing TRG and TC expression of the MRE11 protein (*P* = 0.015, Fig 3B) and of the combined two-protein panel (*P* = 0.011, Fig 3C). The discriminatory power of each protein biomarker and their combination in TC was characterized using a ROC-AUC analysis of good response (TRG 0–2) versus poor response (TRG3) groups. The average ROC-AUC was 0.745 for the combined panel of two proteins, compared with 0.618 for ATM and 0.711 for MRE11 proteins (Fig 3D). These results suggest that the combined two protein biomarkers provide good discrimination between good and poor tumor responses after radiotherapy.

## Discussion

Despite intensive research into biomarkers and prognostic factors for CRC, little is known about the factors influencing the survival of patients with rectal cancer. Although there is overlap with the treatment of colon cancer in the metastatic setting, rectal cancers in the locoregional setting are managed differently from colon cancers [16]. The 1997 Swedish Cancer Trial of patients with resectable rectal cancer showed that preoperative radiotherapy improved both short-term and long-term local recurrence rates and OS compared with surgery alone [17] [18], although the authors noted that the use of radiotherapy was associated with side effects [17]. Since then, the benefits of preoperative radiotherapy or chemoradiotherapy have been corroborated in several meta-analyses [3,4,19,20].

At the individual level however, there can be significant variation in the degree of initial tumour response to radiotherapy. In this context, the TRG score provides an indicator of the regressive response of a tumor to cytotoxic treatment such as radiotherapy. The AJCC Staging Manual (7^th^ ed.) recommends consideration of the TRG independent of the TNM system. The TRG provides important prognostic information because complete or subtotal tumor regression is widely recognized to be associated with better patient outcomes [3,6,19]. Unfortunately, the majority of patients show no response or only a partial response to neoadjuvant therapy. For example, none of the patients in our study showed a complete TRG response; 82.4% showed a poor response and 17.6% showed a minimal to moderate response. However, a meta-analysis performed by Lee et al indicated that even partial tumor regression after preoperative chemoradiotherapy improves DFS, and should be considered as a favorable prognostic factor [21].

Given the dismal prognosis of patients with rectal cancer and the variation in response to radiotherapy, a reliable biomarker of intrinsic tumor radiosensitivity would improve assessment of prognosis and aid in making proper treatment decisions. It would help to avoid adverse side effects of neoadjuvant radiotherapy in patients who are unlikely to benefit, and at the same time identify those who may subsequently benefit from intensification of therapy. Although several biomarkers have been studied as predictors of radiotherapy response [22], the most extensively studied are MRE11 and the radiosensitivity index (RSI), a multigene expression model of tumor radiosensitivity, which was developed to predict treatment outcomes independent of disease site [23]. However, to date, these biomarkers have not been validated in randomized trials, and their clinical value remains unclear. For instance, Sheridan and colleagues [24] found no correlation between MRE11 expression and survival or radiosensitivity in patients with CRC, albeit with the limitations of a small sample size and short follow-up period. In our study, MRE11 expression alone was not significantly associated with any clinicopathologic characteristics, while in terms of ATM only low expression in TP was associated with older age and higher histologic grade, whereas ATM expression in TC was not associated with any clinicopathologic characteristics.

Based on the hypothesis that both ATM and MRE11 are integral to the detection of DNA damage and subsequent intracellular signalling following radiotherapy, and hence their deficiency would equate to increased radiosensitivity, we established a two-marker panel of ATM and MRE11 expression by binary regression analysis of tumor samples and normal tissues. Subsequent analyses showed greater association with general clinicopathologic parameters than either marker alone, as well as yielding a ROC-AUC value of 0.745 in predicting poor histological tumour regression (i.e. TRG 3) following radiotherapy. The latter was superior to using ATM (0.618) or MRE11 (0.711) alone. Furthermore, in both the neoadjuvant radiotherapy sub-cohort and the overall cohort, the combined ATM/MRE11 expression levels correlated with clinical survival outcomes. Thus these markers, when used together, related to both early (histological tumour regression) and late (clinical survival) response to neoadjuvant therapy in rectal cancers. These findings support our hypothesis and are also consistent with the fact that inhibitors of ATM and the MRN complex have been shown to have potential as radiosensitizing agents [25].

It is evident in our results that there are differences in the expression levels and their association with clinicopathological variables and survival outcomes when comparing the results from the centre versus periphery of the tumour mass. Effects of the sampling site on associations with tumor protein expression have been noted previously. For example, in an evaluation of biomarkers in ovarian cancer, Permuth-Wey et al reported sampling variability in protein expression analysis using tissue microarrays [26]. The authors concluded that, for ovarian cancer at least, more reliable data were obtained from the TP than from the TC. This was attributed to optimal exposure to fixatives at the periphery of the processed tissues, although the effect of tumor heterogeneity on sampling variation should also be considered. For instance, TCs in general are more liable to hypoxia and necrosis compared to TP, and ischaemic injury may be another potential source for the biological differences seen within the same tumour.

On a final note, the role of LN status in predicting outcomes is intriguing, and hints at the complexity of using biomarkers to predict patient outcomes. Among patients with LN-negative tumors, there was no significant difference in survival between patients with high vs. low two-protein combined panel score in TC. In contrast, in patients with LN-positive tumors, high two-protein combined panel scores in TC were significantly associated with worse survival. This suggests that the combined expression of ATM and MRE11 may be associated with LN involvement in relation to patient survival.

## Conclusions

Overall, our findings suggest that the optimal management of rectal cancer requires tailored treatment based on biomarker expression such as the two-protein MRE11/ATM panel described here, as well as clinicopathologic characteristics. Further elucidation of biomarkers that predict the response to radiotherapy and/or chemoradiotherapy will constitute important progress toward the individualized treatment of patients with rectal cancer. In this regard, we are continuing studies on ATM and MRE11 in independent datasets focusing on pre-treatment specimens in comparison to post-treatment specimens.

## Supporting Information

S1 Table Performance of two protein classification models. (XLSX)

S2 Table Associations between ATM expression and clinicohistopathological data. (XLSX)

S3 Table Associations between MRE11 expression and clinicopathological data. (XLSX)
